# Polimorfismos del gen de la apolipoproteína E en adultos mayores de 60 años con disminución de la memoria cognitiva y enfermedad de Alzheimer en diferentes poblaciones venezolanas

**DOI:** 10.7705/biomedica.5889

**Published:** 2022-05-01

**Authors:** Silvia Martínez, Bárbara Ochoa, María Rafaela Pérez, Fátima Torrico, Ildemaro García, Carmen Cristina García

**Affiliations:** 1 Facultad de Farmacia, Universidad Central de Venezuela, Caracas, Venezuela Universidad Central de Venezuela Facultad de Farmacia Universidad Central de Venezuela Caracas Venezuela; 2 Instituto de Medicina Experimental, Facultad de Medicina, Universidad Central de Venezuela, Caracas, Venezuela Universidad Central de Venezuela Instituto de Medicina Experimental Facultad de Medicina Universidad Central de Venezuela Caracas Venezuela; 3 Departamento de Cómputo Científico, Universidad Simón Bolívar, Caracas, Venezuela Universidad Simón Bolívar Departamento de Cómputo Científico Universidad Simón Bolívar Caracas Venezuela

**Keywords:** enfermedad de Alzheimer, disfunción cognitiva, demencia, apolipoproteína E4, Venezuela, Alzheimer’s disease, cognitive dysfunction, dementia, apolipoprotein E4, Venezuela

## Abstract

**Introducción.:**

La enfermedad de Alzheimer constituye un problema de salud pública que tiende a agravarse en el tiempo. Entre los factores genéticos de predisposición más importantes, se encuentra la presencia del alelo ε_4_ del gen *APOE* que codifica para la apoproteína E.

**Objetivo.:**

Determinar las frecuencias alélicas y genotípicas de las isoformas de *APOE* en adultos mayores de 60 años con memoria cognitiva disminuida y Alzheimer, en la gran Caracas y en la comunidad indígena pemón de la zona Kamarata-Kanaimö, Estado Bolívar.

**Materiales y métodos.:**

Se estudiaron 267 pacientes: 96 controles, 40 con memoria cognitiva disminuida y 108 con Alzheimer procedentes de Caracas, y 23 individuos de Kamarata-Kanaimö. Las isoformas de *APOE* se determinaron con el estuche AP1210Z: Seeplex ApoE genotyping™.

**Resultados.:**

El alelo ε4 mostró asociación significativa con la memoria cognitiva disminuida (OR=5,03; IC_95%_ 0,98-25,70) y la enfermedad de Alzheimer (OR=5,78; IC_95%_ 1,24-26,85). Las frecuencias genotípicas de los grupos de control y con memoria cognitiva disminuida, fueron: ε3/ε3> ε3/ε4> ε2/ε4> ε3/ε2> ε4/ε4, y las del grupo con Alzheimer: ε3/ε3> ε3/ε4> ε4/ε4> ε2/ε4> ε3/ε2. En Kamarata-Kanaimö, el orden fue ε3/ε3> ε3/ε4> ε4/ε4 y no se encontró el alelo ε2.

**Conclusiones.:**

Las frecuencias alélicas y genotípicas de *APOE* en la muestra tuvieron una distribución similar a la de otros estudios en Venezuela y las Américas. La ausencia del alelo ε2 en la comunidad indígena de Kamarata-Kanaimö amerita mayor investigación. Se constató la asociación positiva del alelo ε4 en personas con la enfermedad de Alzheimer y con memoria cognitiva disminuida. Conocer precozmente los pacientes portadores de este alelo puede ayudar a establecer medidas preventivas en nuestra población.

## Abstract (en)

**Introduction:**

Alzheimer’s disease represents a serious public health problem that tends to worsen over time. Among the most important genetic predisposing factors is the presence of the ε4 allele of the apoprotein E gene (*APOE*).

**Objective:**

To determine the allelic and genotypic frequencies of the *APOE* isoforms in adults over 60 years old with mild cognitive impairment and Alzheimer’s disease in Gran Caracas and in the indigenous Pemón community of the Kamarata-Kanaimö area, Bolívar State.

**Materials and methods:**

We studied 267 patients: 96 controls, 40 with mild cognitive impairment, 108 with Alzheimer’s from Caracas, and 23 individuals from Kamarata-Kanaimö. The *APOE* isoforms were determined with the AP1210Z: Seeplex® ApoE Genotyping kit.

**Results:**

The allele ε4 showed a significant association with mild cognitive impairment (OR=5.03; 95% CI: 0.98-25.70) and EA (OR=5.78; 95% CI: 1.24-26.85). The genotype frequencies for the control and mild cognitive impairment groups were ε3/ε3> ε3/ε4> ε2/ε4> ε3/ε2> ε4/ε4, and for the Alzheimer’s group, ε3/ε3> ε3/ε4> ε4/ε4> ε2/ε4> ε3/ε2 In Kamarata- Kanaimö, the order was ε3/ε3> ε3/ε4> ε4/ε4; the allele ε2 was not found in this group.

**Conclusions:**

*APOE* allelic and genotypic frequencies in our sample showed a similar distribution to those found in other studies in Venezuela and the Americas. The absence of the ε2 allele in the indigenous community of Kamarata-Kanaimö warrants further investigation. The positive association of the ε4 allele with both Alzheimer’s and mild cognitive impairment was reinforced. The early determination of the ε4 allele carriers can help establish preventive measures in our population.

La enfermedad de Alzheimer es una afección de carácter progresivo e irreversible asociada con la neurodegeneración cerebral, que afecta a millones de personas a nivel mundial, usualmente después de los 60 años ^1^. Es la forma más frecuente de demencia, y representa entre 60 y 70 % de los casos a nivel global ^1^.

Actualmente hay 4,1 millones de personas con demencia en Latinoamérica y el Caribe, y se estima que la cifra se incrementará a 9,1 millones en el 2040, es decir, será similar a la de Norteamérica ^2^. Se plantea que en Venezuela 140.000 adultos mayores sufren de la enfermedad ^3^, con una mayor incidencia reportada (12,13 %), en el estado Zulia, seguido por el estado Miranda (11,67 %) y el Distrito Capital (9,91 %) ^3^. En el marco de los países estudiados por el grupo 10/66 *Dementia Research Group* (Cuba, República Dominicana, Perú, México, China y Venezuela), Venezuela registró la mayor incidencia en el grupo de personas mayores de 80 años ^4^. Este dato es de gran importancia, dado que las proyecciones del Instituto Nacional de Estadística ^5^ sugieren que la población de adultos mayores se va a triplicar en 30 años, pasando de 1’991.738 en el 2015 a un estimado de 6’304.070 en el 2045. Este aumento de la población de adultos mayores incidirá directamente en las cifras de demencia del país, lo que tendrá un impacto notable en los servicios de salud, a menos que se puedan desarrollar medidas preventivas eficaces, como el diagnóstico y el tratamiento precoces.

En Venezuela, la enfermedad de Alzheimer fue la vigésima quinta causa de muerte según las estadísticas de mortalidad del 2013 ^6^, pero se ha observado un incremento continuo a lo largo de la última década en la tasa de mortalidad de los pacientes con esta enfermedad. Debe anotarse que las cifras correspondientes a los años posteriores al 2013 no se encuentran disponibles (figura 1).

**Figura 1.**
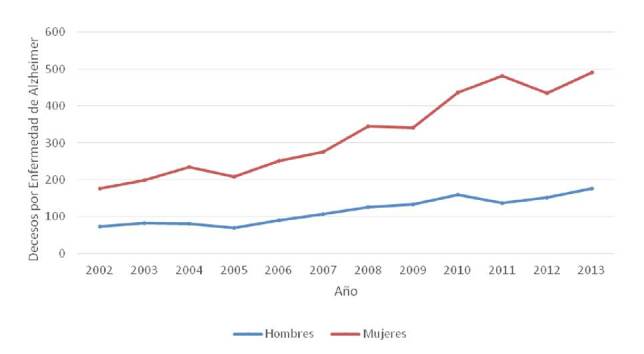
Tasa de mortalidad para la enfermedad de Alzheimer de aparición tardía (>60 años) en Venezuela, 2002-2013 Nota: el anuario de mortalidad del 2013 fue publicado en diciembre de 2015; no hay anuarios publicados que incluyan datos desde el 2014.

La región cerebral más afectada por la enfermedad de Alzheimer es la corteza entorrinal; también, se ha determinado que el hipocampo, perteneciente al sistema límbico, es una de las zonas más vulnerables y exhibe una neuropatología grave en las etapas tempranas de la enfermedad. Una de las funciones afectadas por el daño en el hipocampo es la memoria anterógrada episódica, también conocida como memoria cognitiva. La pérdida de las neuronas en esta zona conlleva la atrofia del tejido, lo que ha sido ampliamente documentado mediante los estudios histológicos *post mortem* y las imágenes diagnósticas ^7^.

El aumento de la esperanza de vida en las poblaciones latinoamericanas lleva a preguntarse si el sistema público de salud estará preparado para detectar a las personas con signos de problemas cognitivos. Como consecuencia de la prolongada fase preclínica de la enfermedad de Alzheimer, hoy se han incrementado los esfuerzos para detectar la disminución de la memoria cognitiva, ya que se la ha propuesto como una fase intermedia entre un individuo sano y aquel con demencia. Los pacientes con esta alteración pueden desarrollar enfermedad de Alzheimer, enfermedad vascular u otros tipos de demencias, o pueden permanecer estables y, en algunos casos, incluso, revertir su condición y recuperar las funciones cognitivas normales ^8^.

El marcador genético más conocido para la enfermedad es el gen *APOE,* ubicado en el cromosoma 19 (q13.2.-q13.3), que codifica para la apoliproteína E (ApoE) constituida por 299 aminoácidos. Se trata de un gen polimórfico con tres alelos conocidos: ε_2_, ε_3_ y ε_4_, los cuales se heredan de manera codominante. Es decir, cada uno de los padres aporta un alelo, lo que resulta en tres genotipos homocigotos: ε2/ε2, ε3/ε3 y ε4/ε4, y tres genotipos heterocigotos: ε2/ε3, ε2/ε4 y ε3/ε4. El alelo ε_3_ corresponde al salvaje, en tanto que los alelos ε2 y ε4 se consideran variantes que se diferencian entre sí únicamente por la sustitución de los aminoácidos de los codones en posición 115 y 158. Cada alelo codifica para una proteína conocida como ApoE ε2, ε3 y ε4 ^9^^,^^10^.

En Venezuela, el Instituto Nacional de Estadística afirma que la población indígena nacional aumentó a 725.141, y que el 7,54 % de ellos se encuentra en el estado Bolívar, incluidos 27.157 pemones ^5^. Entre los pemones, 24.121 se ubican en asentamientos distinguidos como “comunidades indígenas”, una de las cuales es Kamarata-Kanaimó (56°97’55” E y 63°45’02” N), con 605 habitantes ^5^^,^^11^.

Los pemones son indígenas suramericanos que hablan el pemón, perteneciente a la familia de idiomas caribes. Habitan la zona sureste del estado Bolívar en Venezuela, en la frontera con Guyana y Brasil, y están diseminados en el municipio Gran Sabana, que ocupa 32.990 km² con una densidad poblacional de 0,93 habitantes/km². Estas tierras pertenecen al Parque Nacional Canaima y se consideran el hábitat ancestral del pueblo pemón, uno de cuyos asentamientos es la comunidad Kamarata-Kanaimö. El municipio es un parque considerado patrimonio natural protegido, región fronteriza de máxima seguridad, zona de explotación minera, destino turístico de renombre internacional y enlace geográfico con el mayor mercado de América del Sur ^12^.

Hay pocos estudios publicados sobre los polimorfismos de *APOE* en la población venezolana ^13^^-^^15^ y, menos aún, en la población indígena ^13^, por lo que en nuestro trabajo el objetivo fue determinar las frecuencias alélicas y genotípicas de las isoformas del gen *APOE* en adultos mayores de 60 años con disminución de la memoria cognitiva o enfermedad de Alzheimer en la población de la Gran Caracas y en una comunidad indígena pemón de la zona Kamarata-Kanaimö, Estado Bolívar. Dada la precaria situación del sector salud en Venezuela y la creciente población afectada por esta enfermedad, es necesario estudiar la prevalencia de biomarcadores en sangre periférica ya conocidos, que constituyen marcadores de riesgo para su desarrollo.

## Materiales y métodos

### Población de estudio

El diseño experimental abarcó el estudio de 267 muestras distribuidas en 96 controles, 40 pacientes con disminución de la memoria cognitiva diagnosticada por el médico internista o el especialista en neurología, 108 pacientes con enfermedad de Alzheimer diagnosticada por el médico especialista y 23 personas pertenecientes a la comunidad indígena de Kamarata-Kanaimö (56°97’55” E y 63°45’02” N). Todos los individuos involucrados tenían mas de 60 años cumplidos en el momento del estudio. Se hizo la genotipificación para *APOE* en cada uno de los participantes y, posteriormente, el análisis de frecuencias y asociación.

Las muestras de los controles, los pacientes con disminución de la memoria cognitiva y enfermedad de Alzheimer, se tomaron con el consentimiento informado de todos los individuos involucrados o sus representantes. Se contó con la aprobación del Cómite de Ética del Instituto de Medicina Experimental de la Facultad de Medicina de la Universidad Central de Venezuela y del Hospital Clínico Universitario de Caracas.

Los participantes en el estudio fueron evaluados y estratificados por neurólogos clínicos. Para asignarlos a cada grupo, los neurólogos utilizaron el *Mini-Mental Test* (MMT) y una encuesta cognitiva de diagnóstico validada por el grupo 10/66 (Hospital Clínico Universitario de Caracas, UCV). De acuerdo con el puntaje obtenido por cada paciente, se le asignaba al grupo de control, de disminución de la memoria cognitiva o de enfermedad de Alzheimer. Los neurólogos y médicos internistas decidían a cuáles pacientes se les realizaban estudios de imágenes para ayudar a la confirmación de la enfermedad.

La muestra de los individuos de la comunidad indigena de Kamarata- Kanaimö estuvo constituida por hombres y mujeres que no presentaron sintomatología de disminución de la memoria cognitiva o de enfermedad de Alzheimer, y cuyo MMT arrojó resultados normales. El médico asignado al servicio ambulatorio de la zona había sido entrenado para realizar las pruebas cognitivas, y fue el encargado de hacer la evaluación física y con el MMT.

Las instituciones y casas hogares de la Gran Caracas que colaboraron en el presente estudio, fueron el Hospital Clínico Universitario de Caracas, el Hogar Residencial Santo Domingo (Av. Neverí, Colinas de Bello Monte), la Casa Hogar Santa Isabel (Av. Neverí, Colinas de Bello Monte), la Casa Hogar Venezuela SRL (Av. Maracaibo con Av. Maturín, Las Palmas), y el Ancianato Fundación Cristóforo Colombo (Villa Pompei), San Antonio de los Altos, Estado Miranda.

### Análisis molecular

Se extrajeron 12 ml de sangre periférica a los individuos en los establecimientos, instituciones y hogares mencionados, y se los incluyó en cada grupo según los criterios ya expuestos. Las muestras se recolectaron en tubos con EDTA como anticoagulante. Una vez separada la alícuota para la extracción de ADN, las muestras de sangre completa preservadas con EDTA y el ADN aislado (DNAeasy® Blood and Tissue, Quiagen) se identificaron debidamente y se almacenaron a -70 °C.

Para determinar las isoformas de *APOE* se utilizó el kit AP1210Z: Seeplex ApoE Genotyping™, técnica *in vitro* cualitativa para la detección de los genotipos de *APOE* que se basa en la tecnología de cebadores duales (DPO™), facilita la optimización de la reacción en cadena de la polimerasa (PCR), y maximiza la especificidad y la sensibilidad bloqueando la alineación inespecífica de los cebadores. El control interno define una banda de 779 pares de bases (pb). El peso molecular de las bandas según las isoformas de APOE son las siguientes: 158Cys (517 pb), 112Arg (447 pb), 112Cys (376 pb) y 158Arg (311 pb).

El termociclador se precalentó a 94 °C antes de colocar las muestras para comenzar la PCR en caliente. Se utilizó el siguiente programa de amplificación: desnaturalización a 95 °C durante 15 minutos, seguida por 35 ciclos a 94 °C durante 0,5 minutos, a 65 °C durante 0,5 minutos, a 72 °C durante 2 minutos y una última fase de extensión a 72 °C durante 10 minutos.

Un volumen de 5 µl de los productos amplificados se cargó en un gel de agarosa al 2 % con bromuro de etidio y se hizo una electroforesis a 120 V durante 20 minutos.

De acuerdo con el sistema de genotipificación de los polimorfismos de *APOE* rs429358/rs7412 utilizado, los resultados obtenidos de la amplificación fueron los siguientes:

### Análisis estadístico

La razón de momios (*Odds Ratio,* OR) se determinó asumiendo un modelo aditivo donde se estima que cada copia del alelo ε_*4*_ modifica la asociación en forma aditiva. Por lo tanto, los homocigotos ε_4_/ε_4_ tendrían una asociación mayor con la enfermedad que los heterocigotos ε3/ε4, y ε2/ε4. El paquete utilizado fue la función *logit* del programa de uso libre R.

## Resultados

Todos los individuos incluidos en el presente trabajo eran mayores de 60 años: la media de la edad fue de 68,7±8 años en el grupo de control y de 83,59±8 años en el grupo de pacientes con enfermedad de Alzheimer (cuadro 1). Las mujeres representaban el 60 % o más en todos los grupos, con excepción de la comunidad indígena en la que correspondieron al 52 % de los participantes.

**Cuadro 1:** Variables demográficas

Variable demográfica	Control (N=96)	MCD (N=40)	EA (N=108)	Kamarata-Kanaimö (N=23)
Edad (años)	68,7 ± 8	70,5 ± 7,96	83,59 ± 8	68,5 ± 7,5
Mujeres	59 (61 %)	24 (60 %)	79 (73 %)	12 (52 %)
Hombres	37 (39 %)	16 (40 %)	29 (27 %)	11 (48 %)

MCD: memoria cognitiva disminuida; EA: enfermedad de Alzheimer

Los alelos y genotipos de los polimorfismos se distribuyeron mediante conteo directo y se calcularon las respectivas frecuencias (cuadro 2). La distribución de los genotipos en el grupo de control se encontraba en equilibrio de Hardy-Weinberg. El alelo ε3 fue el más frecuente en todos los grupos muestrales, seguido por el alelo ε4. El alelo ε2 estaba presente en los grupos de la Gran Caracas, , pero no así en el grupo de Kamarata-Kanaimö (cuadro 2).

**Cuadro 2:** Frecuencias genotípicas y alélicas de polimorfismos del gen APOE de los grupos muestrales

Grupo	n	Frecuencias genotípicas^a^					Frecuencias alélicas		
		ε3/ε3	ε3/ε4	ε3/ε2	ε4/ε4	ε2/ε4	ε3	ε4	ε2
Control	96	0,57	0,25	0,06	0,01	0,11	0,73	0,18	0,09
Memoria cognitiva disminuida	40	0,53	0,32	0,021	0,08*	0,049	0,70	0,26*	0,04
Enfermedad de Alzheimer	108	0,48	0,36	0,026	0,071*	0,063	0,68	0,24*	0,08
Kamarata-Kanaimőa	23	0,52	0,39	-	0,09	-	0,71	0,29	-

^a^ No se encontró el genotipo ε2/ε2.

MCD: memoria cognitiva disminuida; EA:

^*^ p<0,05 con respecto al grupo de control

En cuanto a las frecuencias genotípicas de los grupos de control y aquellos con disminución de la memoria cognitiva, su orden de mayor a menor fue ε3/ε3> ε3/ε4> ε2/ε4> ε3/ε2> ε4/ε4. No se encontraron portadores homocigotos del genotipo ε2/ε2. En el grupo con enfermedad de Alzheimer, el orden de los genotipos fue ε3/ε3> ε3/ε4> ε4/ε4> ε2/ε4> ε3/ε2; en tanto que, en el grupo de la comunidad Kamarata-Kanaimö, fue ε3/ε3> ε3/ε4> ε4/ε4 y no se encontraron isoformas que incluyeran al alelo ε2 (cuadro 2). Los individuos que presentaban el alelo *APO* ε4 mostraron una asociación positiva entre este y la disminución de la memoria cognitiva (OR=2,29; IC^95%^ 1,3539-3,8893; p<0,02). Asimismo, utilizando el modelo dominante para analizar este polimorfismo, se encontró una asociación cuando los individuos eran portadores homocigotos de ε4/ε4 (OR=5,03; IC_95%_ 0,98-25,70; p<0,05). Además, los individuos con el alelo *APO*ε4 mostraron una asociación positiva entre su presencia y la enfermedad de Alzheimer (OR=1,5248; IC_95%_ 0,9624- 2,4147; p<0,05). Según el modelo dominante, hubo una mayor asociación entre la enfermedad de Alzheimer y los portadores homocigotos de ε4/ε4 (OR=5,78; IC_95%_ 1,24-26,85; p<0,05). En el grupo de la comunidad Kamarata-Kanaimö, no se encontraron individuos con disminución de la memoria cognitiva o enfermedad de Alzheimer.

Al corregir las frecuencias según el sexo, se sugirió una asociación positiva entre el alelo ε4 y la disminución de la memoria cognitiva en los portadores: mujeres (OR=1,8450; IC^95%^ 0,8957-3,8002; n.s. p<0,09) y hombres (OR=1,55; IC_95%_ 0,5577-4,380; n.s. p<0,40). En el cuadro 3, se muestran las frecuencias genotípicas y alélicas discriminadas por sexo en los diferentes grupos muestrales. Al comparar las frecuencias del alelo ε4 en los grupos con enfermedad de Alzheimer y de control, se observaron diferencias estadísticamente significativas en las mujeres con enfermedad de Alzheimer (χ χ^2^=4,53; grados de libertad= 1; α=0,05).

**Cuadro 3:** Frecuencias genotípicas y alélicas de polimorfismos de APOE discriminadas por sexo en los grupos muestrales

Grupo	n	Frecuencias genotípicas^a^					Frecuencias alélicas		
		ε3/ε3	ε3/ε4	ε3/ε2	ε4/ε4	ε2/ε4	ε3	ε4	ε2
Control									
Mujeres	59	0,53	0,33	0,045	0,015	0,076	0,74	0,20	0,06
Hombres	37	0,63	0,20	0,08		0,09	0,76	0,16	0,08
Memoria cognitiva disminuida									
Mujeres	26	0,60	0,30	-	0,03	0,07	0,74	0,22	0,04
Hombres	14	0,47	0,41	0,06	0,06	-	0,70	0,27	0,03
Enfermedad de Alzheimer									
Mujeres	79	0,42	0,39	0,06	0,06	0,07	0,64	0,30	0,06
Hombres	29	0,62	0,24	-	0,11	0,03	0,71	0,28	0,01
Kamarata-Kanaimőa									-
Mujeres	12	0,75	0,25	-	-	-	0,87	0,13	-
Hombres	11	0,54	0,36	-	0,1	-	0,66	0,24	-

^a^ No se encontró el genotipo ε2/ε2

## Discusión

El marcador genético más conocido para la enfermedad de Alzheimer es el gen *APOE*; sin embargo, no todos los casos se pueden explicar por la presencia del alelo ε4, lo que ha llevado a determinar que existen otros genes que pueden contribuir al riesgo de desarrollar la enfermedad.

Martínez, *et al.* (2007), describieron las frecuencias de haplotipos en Caracas analizando el ADN mitocondrial. Encontraron que existía una gran proporción de haplotipos pertenecientes a amerindios, cuyos porcentajes variaban entre 43 y 72 % dependiendo del nivel socioeconómico de la población ^16^. Gómez-Carballa, *et al*. (2012), analizaron los procesos de mezcla racial ocurridos en Venezuela durante los últimos siglos. Se suponía que el componente indígena de la población había desaparecido progresivamente de la Venezuela urbana debido a las sucesivas olas de inmigración provenientes de Europa, África subsahariana y, en tiempos recientes, de países vecinos de América. Sin embargo, encontraron que el componente de ADN mitocondrial proveniente de amerindios era de 65 % en una muestra de la Gran Caracas ^17^. Estos hallazgos nos ayudan a comprender las posibles razones de los resultados de la presente investigación, según los cuales existe una gran variabilidad genética en la distribución de los alelos del gen *APOE* en la población venezolana (cuadro 4).

**Cuadro 4:** Distribuciones alélicas porcentuales de APOE encontradas en el presente estudio y otras investigaciones realizadas en Venezuela

Alelos	Gran Caracas n=96	Kamarata- KanaimŐ, Estado Bolívar n=23	**Molero, *et*** ***al*., 2001** **n=1.665**	**Fernández, *et al*., 2005** **n=40** **Caucásicos**	Fernández, et al., 2005 n=87 Mestizos	Arráiz, et al., 2008 n=88
		Pemones	Estado Zulia	Estado Miranda	Estado Miranda	Estado Zulia
Distribución de porcentajes						
APOE ε2	9	-	5	3,8	18,9	10
APOE ε3	75	80	84	81,2	71,3	78
APOE ε4	16	20	11	15	9,8	12

La primera y la segunda columna muestran los resultados obtenidos en el presente estudio; las columnas 3 a 6 reflejan los resultados de otros estudios en regiones venezolanas.

Nótese la variabilidad de los porcentajes en los diferentes trabajos ^18^^,^^19^^,^^20^.

Pocas comunidades humanas pueden considerarse realmente aislamientos genéticos. Como se ha demostrado en el último siglo, en varias poblaciones existe cierto grado de consanguinidad que no alcanza una magnitud tal que permita clasificarlas como en aislamiento geográfico. Para comprender la mezcla o posible aislamiento de la población indígena venezolana, Merriwether, *et al.* (2000), llevaron a cabo un análisis del ADN mitocondrial de la comunidad Yanomami, la cual habita también en el Estado Bolívar y el norte de Brasil ^18^. Encontraron un gran componente amerindio en los pobladores, con haplotipos D (40 %) y C (51 %). Posteriormente, Williams, *et al*. (2002), encontraron en dicha comunidad un patrón variable de haplotipos: D (12 %), B (56 %) y C (32 %) ^19^

Entre el 2000 y el 2005, se llevaron a cabo análisis de ADN mitocondrial, del cromosoma Y y de polimorfismos autosómicos, en muestras de poblaciones aisladas en Antioquia (Colombia) y en el Valle Central de Costa Rica. Se encontró que ambas comunidades eran genéticamente muy similares, lo cual indica que tienen una relación cercana desde su fundación. En ambas poblaciones, la mayoría de los ancestros eran amerindios, con predominio de europeos entre los hombres y de amerindias entre las mujeres ^20^^,^^21^. Estos procesos raciales no se han investigado en el pueblo pemón, por lo que solo se puede suponer que los patrones de mezcla de estos indígenas pueden ser similares a los observados en comunidades cercanas y nos permitirían explicar la distribución alélica encontrada del gen *APOE* (cuadro 4). Además, puede haber influencia de ancestros masculinos europeos o subsaharianos en la composición genética de esta población indígena.

### Distribución del alelo ε3

El ε_3_ se considera como el alelo silvestre en la mayoría de la población mundial y su expresión está involucrada en la homeostasis lipídica y en el sistema nervioso central con la captación y remoción del β-amiloide. Su expresión en homocigosis o heterocigosis permite que la proteína ApoE, mediante su función de distribuir los lípidos en las células de la microglía, sea capaz de remover las fracciones de β-amiloide para que no haya dimerización y formación de cúmulos de proteína amiloide, característicos de enfermedad de Alzheimer ^22^. En el presente estudio, se encontró una distribución alélica de 75 % en el grupo control y de 80 % en la comunidad indígena de Kamarata-Kanaimö. Estos resultados son cercanos a los de otros trabajos de investigación sobre los genotipos de *APOE* realizados en Venezuela (cuadro 4). Estos estudios se centraron en las poblaciones del Estado Zulia, donde se observaron distribuciones alélicas de 84 % ^14^ y 78% ^15^, respectivamente; además, el de Fernández, *et al,* (2005), que encontraron 71,3 % en la Gran Caracas y 81,2 % en el Estado Miranda ^13^.

En trabajos realizados en ciudades de Colombia, se reportó el alelo ε3 con una frecuencia de 92 % en Medellín, de 85 % en Barranquilla y de 85,6 % en Bogotá ^23^^,^^24^. Asimismo, otros estudios en Perú y Brasil reportan la presencia del alelo ε3 en frecuencias de 93,9 % y 80 % ^25^^,^^26^. Las diferencias regionales pueden deberse a las composiciones étnicas de cada zona; por ejemplo, de acuerdo con el Censo General 2005, Perfil Barranquilla-Atlántico realizado por el Departamento Administrativo Nacional de Estadística (DANE) de Colombia, 13,2 % de la población de Barranquilla se auto reconoce como negra y 0,1 % indígena. El resto lo constituyen principalmente las razas caucásica y mestiza (Departamento Administrativo Nacional de Estadística (DANE): Censo General 2005. Perfil Barranquilla- Atlántico. Fecha de consulta: 30 de Julio de 2018. Disponible en: http://www.dane.gov.co/files/censo2005/perfiles/atlantico/barranquilla.pdf. También en: https://www.minsalud.gov.co/plandecenal/mapa/analisis-de-Situacion-Salud- Barranquilla-2012-2015.pdf).

### Distribución del alelo ε2

En el presente estudio, el alelo ε2 se distribuyó en el 9 % del grupo control, lo cual coincide con otros trabajos venezolanos de investigación sobre los genotipos del *APOE* (cuadro 3). En dos poblaciones del Estado Zulia, se observaron distribuciones alélicas de 5 % y 10 %, respectivamente ^14^^,^^15^; además, Fernández, *et al.* (2005), encontraron 3,7 % en la Gran Caracas y 18,9 % en el Estado Miranda ^13^.

En Suramérica, se reportó el alelo ε3 en tres ciudades de Colombia, con una frecuencia de 92 % en Medellín, de 85 % en Barranquilla y de 85,6 % en Bogotá ^23^^,^^24^; y además, de 1,1 % en Perú ^25^ y de 6 % en Brasil ^26^.

Aunque el alelo ε2 se encuentra en la mayoría de las poblaciones estudiadas previamente, en el presente estudio no se evidenció en la comunidad indígena de Kamarata-Kanaimö. Esto plantea interrogantes sobre la composición genética de dicha comunidad y el efecto de la ausencia de dicho alelo en la aparición de problemas cognitivos y enfermedad de Alzheimer. Este resultado coincide con lo reportado por Fernández, *et al*. (2005), en las poblaciones amerindias Yucpa y Bari, habitantes de la sierra del Perijá en la frontera colombo-venezolana ^13^.

Se considera que el alelo ε_2_ juega un papel protector frente a la disminución de lipoproteínas de baja densidad y se asocia con la aparición de la hiperlipidemia de tipo III, puesto que la proteína codificada por este alelo no se une con facilidad al receptor hepático, lo que afecta el metabolismo de los quilomicrones asociados con enfermedades metabólicas. Además, se ha reportado que la probabilidad de que un individuo con el genotipo ε2/ε2 sufra la enfermedad de Alzheimer es muy baja ^27^; por lo tanto, este alelo podría tener un efecto neuroprotector ^28^.

En el presente trabajo, la frecuencia de este alelo en la población urbana de la Gran Caracas es alta (9 %), cercana al 10 % encontrado por Arráiz, *et al.* (2008), en una población mestiza del Estado Miranda ^15^. Sin embargo, ambas son altas al contrastarlas con aquellas encontradas en otros trabajos, como el de Molero, *et al.* (2001), donde se reporta una frecuencia de 5% ^14^, comparable al 3,8% informada por Fernández *et al.* (2005) ^13^.

La gran frecuencia del alelo ε3 puede interpretarse fisiológicamente como una disminución en la predisposición para el desarrollo de la enfermedad de Alzheimer. Es de resaltar que, en el grupo con disminución de la memoria cognitiva y en el de enfermedad de Alzheimer, se observó una frecuencia menor del alelo ε2 a la observada en el grupo control, 3 % y 5 %, respectivamente, lo que sugiere que estos individuos serían más proclives a sufrir una de estas dos condiciones.

### Distribución del alelo ε4

Funcionalmente, la isoforma ApoE ε4 presenta una afinidad disminuida por el receptor de lipoproteínas de baja densidad y por las partículas de lipoproteínas, lo cual disminuye la captación de monómeros de β amiloide y su subsecuente remoción por parte de la microglía ^9^^,^^10^.

El alelo ε4 presentó una distribución porcentual de 16 % en la muestra de la Gran Caracas y, una aún mayor (20 %), en la comunidad Kamarata-Kanaimö, porcentajes que resultan altos al compararlos con los de otros trabajos realizados en Venezuela. Los estudios de Molero, *et al.* (2001), y Arráiz, *et al.* (2008), se han centrado en las poblaciones del Estado Zulia, donde observaron frecuencias de 11 % y 12 %, respectivamente ^14^^,^^15^. Por otra parte, Fernández *et al*. (2008), describen frecuencias de 15 % en la Gran Caracas y de 9,8 % en el Estado Miranda ^13^. Además, en la población indígena Warao, nativos del Estado Delta Amacuro, la frecuencia encontrada fue de 11 % ^29^.

Respecto a otros trabajos en el continente americano, en Colombia, se reportan frecuencias para este alelo de 4,1 %, 12,5 % y 13 %, en las ciudades de Medellín, Barranquilla y Bogotá, respectivamente ^23^^,^^24^; mientras que, en Perú, se ha reportado una frecuencia de 5 % ^25^.

Se obtuvo una asociación significativa para el alelo ε4, tanto en el grupo con disminución de la memoria cognitiva (OR=5,03; IC_95%_ 0,98-25,70) como en el de enfermedad de Alzheimer (OR=5,78; IC_95%_ 1,24-26,85); es decir, que la presencia del alelo ε4 aumenta en cinco veces la posibilidad de desarrollar problemas de cognición y, casi en seis, el riesgo de desarrollar enfermedad de Alzheimer. Como se ha subrayado durante este trabajo, la disminución de la memoria cognitiva es una fase transicional entre el envejecimiento normal y la demencia, y se asocia con un mayor riesgo de desarrollar la enfermedad de Alzheimer. La tasa a la que los casos de disminución de la memoria cognitiva progresan a enfermedad de Alzheimer es de 10 a 15 % por año, en contraste con una tasa de 1 a 2 % por año en individuos mayores sanos.

La prevalencia de *APOE* ε4 es mayor en pacientes con disminución de la memoria cognitiva que en adultos mayores sanos y así lo reflejan los resultados obtenidos, tanto así, que los portadores presentan rasgos cognitivos comparables con aquellos que se observan en los estadios tempranos de la enfermedad de Alzheimer.

Un estudio caso-control en exámenes de memoria en portadores del *APOE* ε4 reportó resultados bajos comparados con aquellos que no lo son. La presencia de este alelo se encuentra asociada con memoria ejecutoria disminuida, gravedad y aumento de esta pérdida de memoria a una edad media (40-59 años) y en adultos mayores (60-85 años); además, esta asociación está relacionada con la ¿dosis? de *APOE* ε4 que porte el individuo ^30^.

En estudios prospectivos, se han analizado el efecto del *APOE* y del sexo en la conversión clínica de individuos sanos a pacientes con disminución de la memoria cognitiva y enfermedad de Alzheimer. Se encontró que las mujeres portadoras del alelo *APOE* ε4 fueron más propensas a desarrollar disminución de la memoria cognitiva o enfermedad de Alzheimer, en un intervalo de cuatro años aproximadamente ^31^^,^^32^. Estos trabajos soportan los hallazgos de la presente investigación con respecto a dicho mayor riesgo en mujeres portadoras del alelo ε4.

### Distribución de genotipos del APOE

En el presente estudio, los individuos homocigotos para el alelo ε4 con disminución de la memoria cognitiva, presentaron una asociación significativa y con riesgo de ser perjudicial (OR=5,03; IC_95%_ 0,98-25,70; p<0,05). Así mismo, el valor de OR en el grupo con enfermedad de Alzheimer sugiere un efecto importante de este alelo en homocigosis sobre la predisposición a desarrollar enfermedad de Alzheimer (OR=5,78; IC_95%_ 1,24-26,85; p<0,05). Los resultados coinciden con los de estudios poblacionales en los cuales la asociación entre *APOE* ε4 y la enfermedad de Alzheimer es menor entre afroamericanos (ε4 /ε4, OR=5,7) e hispanos (ε4 /ε4, OR=2,2). Esa asociación mostró ser más fuerte en poblaciones japonesas (ε4 /ε4, OR=33,1) y caucásicas (ε4 /ε4, OR=14,9) ^30^. El *APOE* ε4 está asociado a una mayor prevalencia de enfermedad de Alzheimer y una menor edad de comienzo de la misma. Su frecuencia y la edad media de su comienzo clínico son de 91 % y 68 años en homocigotos ε4/ε4, de 47 % y 76 años en heterocigotos ε2/ε4, ε3/ε4, y de 20 % y 84 años en aquellos individuos que no presentan el alelo ε4. Estos datos indican que la presencia de *APOE* ε4 incrementa el riesgo de desarrollar la enfermedad a una edad temprana, de una forma que depende de los alelos de *APOE* presentes en el genoma del individuo ^30^.

En la comunidad Kamarata-Kanaimö no se encontró ningún caso de demencia y el único individuo (9 %) que presentó el genotipo ε4/ε4 tenía 94 años, por lo que este alto porcentaje puede deberse al tamaño de la muestra estudiada (cuadro 5). Dozzi, *et al.* (2014), describieron una prevalencia de demencia de 4,9 % en comunidades indígenas de la Amazonía brasileña, que tenían una media de edad de 62,3 años ^33^. Esta población era considerada homogénea, y geográficamente aislada, y se logró muestrear a más del 50 % de sus miembros. Todos los participantes presentaban un nivel educativo muy bajo, así como colesterol bajo y tensión arterial baja, características que han sido reconocidas como factores de riesgo para el desarrollo de las demencias de tipo Alzheimer. Caixeta, en un trabajo presentado como póster en el 2011, reportó los hallazgos de otra población de la Amazonía brasileña, en la cual encontró una prevalencia de demencia de 6,4 % y una media de edad de 72,4 años ^34^. En ambos estudios, los autores afirmaron que el bajo riesgo cardiovascular era protector en estas comunidades, aun cuando los niveles educativos eran bajos. Sin embargo, es oportuno comentar que este nivel educativo fue medido desde la perspectiva occidental, sin tomar en cuenta la educación recibida realmente por esas comunidades. Moreno, *et al.* (2017), estimaron la influencia genética ancestral en individuos con enfermedad de Alzheimer de aparición tardía, comparándola con controles, y encontraron que aquellos que tenían ancestros amerindios presentaban un riesgo menor de desarrollarla ^35^. En dicho trabajo se controlaron factores como edad, sexo, *APOE*, educación y estatus socioeconómico, pero no así factores de riesgo cardiovasculares. Si pertenecer a la etnia amerindia supone un factor de protección en el desarrollo de enfermedad de Alzheimer de comienzo tardío, es un aspecto importante que requiere mayor investigación.

**Cuadro 5:** Distribuciones porcentuales de genotipos de *APOE* encontrados en el presente trabajo y otros estudios realizados en Venezuela

Genotipos	Gran Caracas n=96	Kamarata- KanaimŐ, Estado Bolívar n=23	**Molero, *et al*., 2001 n=1665**	**Fernández, *et al*., 2005 n=40**	**Fernández, *et al*., 2005 n=87**	**Arráiz, *et al*., 2008 n=88**
		Pemones	Estado Zulia	Caucásicos Estado Miranda	Mestizos Estado Miranda	Estado Zulia
ε2/ε2	0	0	3	0	0	2,7
ε2/ε3	6	0	8,1	7,5	31	15,9
ε2/ε4	5	0	1	0	7	0
ε3/ε3	57	52	71,3	65	53	57,95
ε3/ε4	25	39	17,9	25	6	23,86
ε4/ε4	1	9	1,4	2,5	3	0

La primera y segunda columnas muestran los resultados obtenidos en el presente estudio; las columnas 3 a 6 reflejan los resultados de otros estudios en regiones venezolanas.

Nótese la variabilidad de los porcentajes en los diferentes trabajos ^18^^,^^19^^,^^20^.

## Conclusión

Las frecuencias alélicas y genotípicas del gen *APOE* en la muestra de adultos mayores de 60 años en la Gran Caracas, mostraron una distribución similar a la de otros trabajos en población venezolana y fueron menores que aquellas reportadas en poblaciones suramericanas, lo cual corrobora su caracterización como factor de riesgo para el desarrollo de disminución de la memoria cognitiva y enfermedad de Alzheimer.

La distribución genotípica del gen *APOE* en la población estudiada mantiene la misma tendencia existente en otros estudios en Venezuela y en otras zonas de las Américas. Sin embargo, la ausencia del alelo ε2 en la comunidad indígena de Kamarata-Kanaimö amerita mayor investigación.

Los individuos homocigotos para el alelo ε4 y con disminución de la memoria cognitiva, presentaron una asociación significativa y perjudicial (OR= 5,03; IC_95%_ 0,98-25,70; p<0,05). Así mismo, el valor de OR en el grupo con enfermedad de Alzheimer sugiere un efecto importante de la presencia del alelo ε*4* en homocigosis sobre la predisposición a desarrollar esta enfermedad (OR=5,78; IC_95%_ 1,24-26,85; p<0,05). El riesgo de sufrir disminución de la memoria cognitiva y enfermedad de Alzheimer en presencia de al menos un alelo ε4, se comprobó en el presente estudio en población de la Gran Caracas, lo cual coincide con los resultados de otros estudios realizados en los Estados Miranda y Zulia.

Se requiere compresión o conocimiento claro de la estructura genética de las comunidades indígenas de Venezuela, para explicar el origen de ciertas enfermedades genéticas y sus implicaciones en el sistema de salud.
